# 3,4-Dimeth­oxy-*N*-(3-nitro­benzyl­idene)aniline

**DOI:** 10.1107/S1600536808034193

**Published:** 2008-10-22

**Authors:** Mehmet Akkurt, Ali Asghar Jarrahpour, Malihe Aye, Mustafa Gençaslan, Orhan Büyükgüngör

**Affiliations:** a Department of Physics, Faculty of Arts and Sciences, Erciyes University, 38039 Kayseri, Turkey; b Department of Chemistry, College of Sciences, Shiraz University, 71454 Shiraz, Iran; c Department of Physics, Faculty of Arts and Sciences, Ondokuz Mayıs University, 55139 Samsun, Turkey

## Abstract

The title compound, C_15_H_14_N_2_O_4_, has two crystallographically independent mol­ecules in the asymmetric unit. In both mol­ecules, the nitro and the two meth­oxy substituents are coplanar with the benzene rings to which they are attached. The benzene rings are nearly coplanar, with dihedral angles between the two benzene rings of 10.39 (8) and 5.95 (8)° in the two mol­ecules. The two independent mol­ecules in the asymmetric unit are rotated with respect to each other such that the dihedral angles between equivalent benzene rings are 49.11 (8) and 63.93 (8)°. In the crystal structure, inter­molecular C—H⋯O hydrogen-bond contacts and a weak C—H⋯π inter­action are observed.

## Related literature

For general background, see: Arora *et al.* (2002 ▶); Desai *et al.* (2001 ▶); El-masry *et al.* (2000 ▶); Jarrahpour & Khalili (2006 ▶); Jarrahpour *et al.* (2004 ▶); More *et al.* (2001 ▶); Phatak *et al.* (2000 ▶); Samadhiya & Halve (2001 ▶); Singh & Dash (1988 ▶); Tanaka & Shiraishi (2000 ▶). For related structures, see: Akkurt *et al.* (2005 ▶, 2008 ▶).
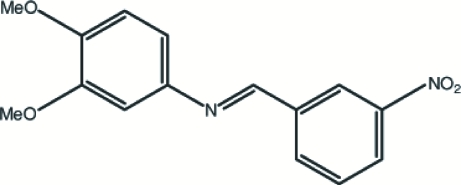


## Experimental

### 

#### Crystal data

C_15_H_14_N_2_O_4_
*M* *_r_* = 286.28
Triclinic, 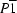

*a* = 8.6345 (8) Å
*b* = 8.6540 (8) Å
*c* = 19.2304 (17) Å
α = 96.629 (7)°
β = 97.338 (7)°
γ = 102.075 (7)°
*V* = 1378.6 (2) Å^3^
*Z* = 4
Mo *K*α radiation
μ = 0.10 mm^−1^
*T* = 296 K
0.78 × 0.36 × 0.07 mm

#### Data collection

Stoe IPDS II diffractometer
Absorption correction: integration (*X-RED32*; Stoe & Cie, 2002 ▶) *T*_min_ = 0.925, *T*_max_ = 0.993
21166 measured reflections
6330 independent reflections
3703 reflections with *I* > 2σ(*I*)
*R*_int_ = 0.065

#### Refinement

*R*[*F* ^2^ > 2σ(*F* ^2^)] = 0.045
*wR*(*F* ^2^) = 0.119
*S* = 0.98
6330 reflections
380 parameters
H-atom parameters constrained
Δρ_max_ = 0.15 e Å^−3^
Δρ_min_ = −0.13 e Å^−3^

### 

Data collection: *X-AREA* (Stoe & Cie, 2002 ▶); cell refinement: *X-AREA*; data reduction: *X-RED32* (Stoe & Cie, 2002 ▶); program(s) used to solve structure: *SIR97* (Altomare *et al.*, 1999 ▶); program(s) used to refine structure: *SHELXL97* (Sheldrick, 2008 ▶); molecular graphics: *ORTEP-3* (Farrugia, 1997 ▶); software used to prepare material for publication: *WinGX* (Farrugia, 1999 ▶).

## Supplementary Material

Crystal structure: contains datablocks global, I. DOI: 10.1107/S1600536808034193/ez2145sup1.cif

Structure factors: contains datablocks I. DOI: 10.1107/S1600536808034193/ez2145Isup2.hkl

Additional supplementary materials:  crystallographic information; 3D view; checkCIF report

## Figures and Tables

**Table 1:** Hydrogen-bond geometry (Å, °)

*D*—H⋯*A*	*D*—H	H⋯*A*	*D*⋯*A*	*D*—H⋯*A*
C8—H8*A*⋯O7^i^	0.96	2.39	3.268 (3)	152
C14—H14⋯O5^ii^	0.93	2.55	3.206 (2)	128
C22—H22*A*⋯O1^iii^	0.96	2.60	3.410 (3)	143
C28—H28⋯O4^iv^	0.93	2.57	3.222 (2)	128
C18—H18⋯*Cg*1	0.93	2.89	3.654 (2)	141

Symmetry codes: (i) 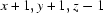; (ii) 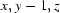; (iii) 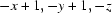; (iv) 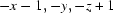. *Cg*1 is the centroid of the C10–C15 ring.

**Fig. 1.**
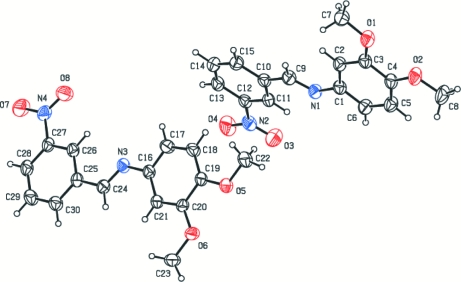
A view of the two independent molecules in the asymmetric unit of the title compound, with the atom-numbering scheme and 30% probability displacement ellipsoids.

**Fig. 2.**
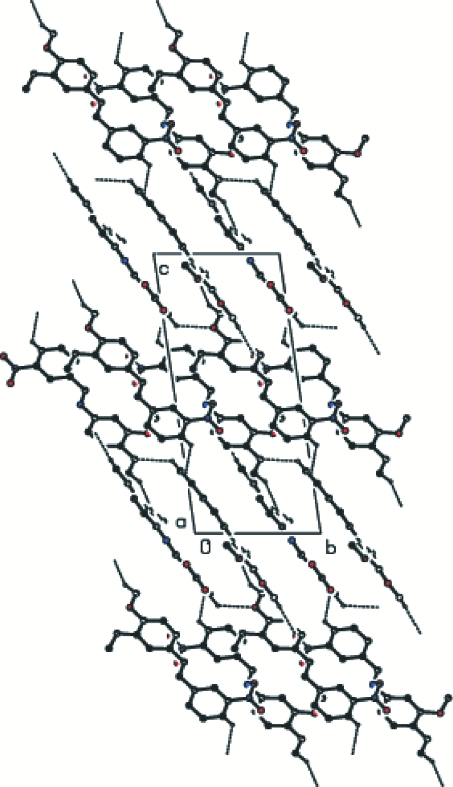
The packing and hydrogen contacts of (I) in the unitcell, down a axis. H atoms not involved in hydrogen bonding have been omitted, for clarity.

## supplementary crystallographic information

### Comment

Schiff bases are widely used for synthetic purposes both by organic and inorganic chemists (Arora *et al.*, 2002) and have uses as biological, analytical, polymer and liquid crystalline materials (Tanaka & Shiraishi, 2000). Schiff bases are reported to show a variety of biological activities such as antibacterial (Jarrahpour & Khalili, 2006; Jarrahpour *et al.*, 2004; El-masry *et al.*, 2000), antifungal (More *et al.*, 2001; Singh & Dash, 1988), anticancer (Desai *et al.*, 2001; Phatak *et al.*, 2000) and herbicidal activities (Samadhiya & Halve, 2001). As an extension of our work on Schiff bases, we report here the crystal structure of the title compound (I).

The two molecules of (I) in the asymetric unit are shown in Fig. 1. In both molecules, the NO_2_ and the two –OCH_3_ substituents are coplanar with the benzene rings and the dihedral angles between the two benzene rings are 10.39 (8)° for C1–C6 and C10–C15 in molecule 1, and 5.95 (8)° for C16–C21 and C25–C30 in molecule 2. The dihedral angles between tequivalent benzene rings in the two independent molecules in the asymmetric unit are 49.11 (8)° for C1–C6 and C16–C21, and 63.93 (8)° for C10–C15 and C25–C30.

In the crystal structure, the packing is stabilized by intermolecular C—H···O type hydrogen contacts and a weak C—H···π interaction (Table 1 and Fig. 2).

### Experimental

A mixture of 3,4-dimethoxyaniline (3 mmol) and 3-nitrobenzaldehyde(3 mmol) was refluxed in EtOH for 4 h. After cooling the solution, the precipitate formed was filtered off and washed with ethanol to give the pure Schiff base as a dark yellow solid [yield 75%, m.p. 389–391 K]. IR(KBr) (cm^-1^) 1616.2 (C═N). ^1^H-NMR (CDCl_3_) δ (p.p.m.) 3.92,3.94 (2 OCH_3_, 2 s, 6H), 6.87–8.72 (ArH, m, 7H), 8.89 (HC═N, s, 1H). ^13^C-NMR (CDCl_3_) δ (p.p.m.) 55.96, 56.11 (2 OCH_3_), 105.62–149.47 (C=C aromatic carbons), 154.97 (C═N).

### Refinement

All H atoms were positioned geometrically, with C—H = 0.93 and 0.96 Å for aromatic and methyl H, respectively, and constrained to ride on their parent atoms with *U*_iso_(H) = 1.2 or 1.5*U*_eq_(C).

### Crystal data

**Table:**

C_15_H_14_N_2_O_4_	*Z* = 4
*M**_r_* = 286.28	*F*(000) = 600
Triclinic, *P*1	*D*_x_ = 1.379 Mg m^−^^3^
Hall symbol: -P 1	Mo *K*α radiation, λ = 0.71073 Å
*a* = 8.6345 (8) Å	Cell parameters from 17758 reflections
*b* = 8.6540 (8) Å	θ = 2.2–28.0°
*c* = 19.2304 (17) Å	µ = 0.10 mm^−^^1^
α = 96.629 (7)°	*T* = 296 K
β = 97.338 (7)°	Plate, yellow
γ = 102.075 (7)°	0.78 × 0.36 × 0.07 mm
*V* = 1378.6 (2) Å^3^	

### Data collection

**Table:**

Stoe IPDS II diffractometer	6330 independent reflections
Radiation source: sealed X-ray tube, 12 x 0.4 mm long-fine focus	3703 reflections with *I* > 2σ(*I*)
plane graphite	*R*_int_ = 0.065
Detector resolution: 6.67 pixels mm^-1^	θ_max_ = 27.6°, θ_min_ = 2.2°
ω scans	*h* = −11→11
Absorption correction: integration (X-RED32; Stoe & Cie, 2002)	*k* = −11→11
*T*_min_ = 0.925, *T*_max_ = 0.993	*l* = −24→24
21166 measured reflections	

### Refinement

**Table:**

Refinement on *F*^2^	Secondary atom site location: difference Fourier map
Least-squares matrix: full	Hydrogen site location: inferred from neighbouring sites
*R*[*F*^2^ > 2σ(*F*^2^)] = 0.045	H-atom parameters constrained
*wR*(*F*^2^) = 0.119	*w* = 1/[σ^2^(*F*_o_^2^) + (0.0559*P*)^2^] where *P* = (*F*_o_^2^ + 2*F*_c_^2^)/3
*S* = 0.98	(Δ/σ)_max_ = 0.001
6330 reflections	Δρ_max_ = 0.15 e Å^−^^3^
380 parameters	Δρ_min_ = −0.13 e Å^−^^3^
0 restraints	Extinction correction: SHELXL97 (Sheldrick, 2008), Fc^*^=kFc[1+0.001Fc^2^λ^3^/sin(2Θ)]^-1/4^
Primary atom site location: structure-invariant direct methods	Extinction coefficient: 0.0065 (13)

### Special details

**Table:**

Geometry. Bond distances, angles *etc*. have been calculated using the rounded fractional coordinates. All su's are estimated from the variances of the (full) variance-covariance matrix. The cell e.s.d.'s are taken into account in the estimation of distances, angles and torsion angles
Refinement. Refinement on *F*^2^ for ALL reflections except those flagged by the user for potential systematic errors. Weighted *R*-factors *wR* and all goodnesses of fit *S* are based on *F*^2^, conventional *R*-factors *R* are based on *F*, with *F* set to zero for negative *F*^2^. The observed criterion of *F*^2^ > σ(*F*^2^) is used only for calculating -*R*-factor-obs *etc*. and is not relevant to the choice of reflections for refinement. *R*-factors based on *F*^2^ are statistically about twice as large as those based on *F*, and *R*-factors based on ALL data will be even larger.

### Fractional atomic coordinates and isotropic or equivalent isotropic displacement parameters (Å^2^)

**Table:**

	*x*	*y*	*z*	*U*_iso_*/*U*_eq_	
O1	0.62826 (14)	0.32088 (17)	−0.08996 (7)	0.0729 (5)	
O2	0.49739 (15)	0.47609 (18)	−0.17819 (7)	0.0779 (5)	
O3	−0.37252 (16)	0.1054 (2)	0.11097 (9)	0.0897 (6)	
O4	−0.39393 (17)	−0.0126 (2)	0.20230 (8)	0.0972 (6)	
N1	0.14725 (16)	0.23760 (17)	0.02524 (8)	0.0601 (5)	
N2	−0.31661 (18)	0.04278 (19)	0.15877 (9)	0.0664 (6)	
C1	0.24228 (19)	0.3003 (2)	−0.02440 (9)	0.0560 (6)	
C2	0.39725 (19)	0.2781 (2)	−0.02885 (9)	0.0572 (6)	
C3	0.47868 (19)	0.3383 (2)	−0.08055 (9)	0.0568 (6)	
C4	0.4075 (2)	0.4223 (2)	−0.12873 (9)	0.0586 (6)	
C5	0.2561 (2)	0.4444 (2)	−0.12409 (10)	0.0667 (7)	
C6	0.1746 (2)	0.3826 (2)	−0.07202 (10)	0.0644 (6)	
C7	0.7050 (2)	0.2369 (3)	−0.04221 (11)	0.0732 (7)	
C8	0.4271 (3)	0.5528 (3)	−0.23003 (13)	0.0983 (10)	
C9	0.2050 (2)	0.1789 (2)	0.07672 (10)	0.0599 (6)	
C10	0.10483 (19)	0.1058 (2)	0.12568 (9)	0.0536 (6)	
C11	−0.05710 (19)	0.10691 (19)	0.11873 (9)	0.0525 (5)	
C12	−0.14762 (19)	0.03390 (19)	0.16476 (9)	0.0529 (5)	
C13	−0.0856 (2)	−0.0437 (2)	0.21671 (9)	0.0604 (6)	
C14	0.0736 (2)	−0.0444 (2)	0.22346 (10)	0.0675 (7)	
C15	0.1685 (2)	0.0304 (2)	0.17885 (10)	0.0630 (6)	
O5	0.06734 (14)	0.60630 (14)	0.26497 (6)	0.0629 (4)	
O6	−0.07174 (16)	0.73981 (14)	0.35747 (7)	0.0704 (5)	
O7	−0.4307 (2)	−0.31605 (19)	0.63291 (10)	0.1093 (7)	
O8	−0.3373 (2)	−0.29259 (17)	0.53686 (9)	0.0982 (7)	
N3	−0.19109 (17)	0.23924 (16)	0.45961 (7)	0.0581 (5)	
N4	−0.38348 (19)	−0.23626 (19)	0.58865 (9)	0.0690 (6)	
C16	−0.1260 (2)	0.33841 (18)	0.41193 (9)	0.0528 (5)	
C17	−0.0490 (2)	0.2699 (2)	0.36272 (10)	0.0694 (7)	
C18	0.0180 (2)	0.3553 (2)	0.31274 (10)	0.0655 (7)	
C19	0.00864 (19)	0.51125 (19)	0.31179 (8)	0.0516 (5)	
C20	−0.06863 (19)	0.58383 (18)	0.36230 (9)	0.0505 (5)	
C21	−0.13542 (19)	0.49789 (19)	0.41131 (9)	0.0526 (5)	
C22	0.1519 (3)	0.5404 (2)	0.21425 (11)	0.0764 (8)	
C23	−0.1356 (3)	0.8214 (2)	0.41150 (12)	0.0798 (8)	
C24	−0.2518 (2)	0.2938 (2)	0.51111 (9)	0.0558 (6)	
C25	−0.31981 (19)	0.18957 (19)	0.56009 (9)	0.0526 (5)	
C26	−0.31842 (19)	0.02855 (19)	0.55182 (9)	0.0538 (5)	
C27	−0.38451 (19)	−0.0654 (2)	0.59839 (9)	0.0537 (5)	
C28	−0.4530 (2)	−0.0053 (2)	0.65350 (9)	0.0631 (7)	
C29	−0.4539 (2)	0.1540 (2)	0.66146 (10)	0.0702 (7)	
C30	−0.3882 (2)	0.2513 (2)	0.61519 (10)	0.0633 (6)	
H2	0.44510	0.22270	0.00310	0.0690*	
H5	0.20820	0.50050	−0.15570	0.0800*	
H6	0.07180	0.39750	−0.06940	0.0770*	
H7A	0.80890	0.23260	−0.05400	0.1100*	
H7B	0.64150	0.13030	−0.04560	0.1100*	
H7C	0.71650	0.29090	0.00530	0.1100*	
H8A	0.50210	0.58450	−0.26140	0.1470*	
H8B	0.39860	0.64560	−0.20760	0.1470*	
H8C	0.33270	0.48090	−0.25660	0.1470*	
H9	0.31420	0.18190	0.08390	0.0720*	
H11	−0.10340	0.15610	0.08360	0.0630*	
H13	−0.15050	−0.09410	0.24630	0.0730*	
H14	0.11830	−0.09540	0.25820	0.0810*	
H15	0.27710	0.03020	0.18450	0.0760*	
H17	−0.04150	0.16430	0.36280	0.0830*	
H18	0.06930	0.30650	0.27980	0.0790*	
H21	−0.18720	0.54610	0.44430	0.0630*	
H22A	0.18730	0.61850	0.18460	0.1150*	
H22B	0.08220	0.44770	0.18560	0.1150*	
H22C	0.24320	0.51080	0.23850	0.1150*	
H23A	−0.13170	0.92910	0.40280	0.1200*	
H23B	−0.07340	0.82270	0.45670	0.1200*	
H23C	−0.24480	0.76760	0.41140	0.1200*	
H24	−0.25330	0.40160	0.51800	0.0670*	
H26	−0.27330	−0.01550	0.51520	0.0650*	
H28	−0.49730	−0.07100	0.68430	0.0760*	
H29	−0.49910	0.19730	0.69830	0.0840*	
H30	−0.39020	0.35900	0.62120	0.0760*	

### Atomic displacement parameters (Å^2^)

**Table:**

	*U*^11^	*U*^22^	*U*^33^	*U*^12^	*U*^13^	*U*^23^
O1	0.0571 (7)	0.1085 (10)	0.0671 (8)	0.0268 (7)	0.0286 (6)	0.0354 (7)
O2	0.0699 (8)	0.1108 (11)	0.0679 (8)	0.0238 (7)	0.0317 (7)	0.0455 (8)
O3	0.0671 (8)	0.1186 (12)	0.1004 (12)	0.0323 (8)	0.0288 (8)	0.0475 (10)
O4	0.0799 (9)	0.1330 (13)	0.0907 (11)	0.0167 (9)	0.0519 (9)	0.0366 (10)
N1	0.0573 (8)	0.0702 (9)	0.0606 (9)	0.0152 (7)	0.0256 (7)	0.0221 (7)
N2	0.0637 (9)	0.0753 (10)	0.0642 (10)	0.0117 (8)	0.0301 (8)	0.0121 (8)
C1	0.0561 (9)	0.0598 (10)	0.0555 (10)	0.0099 (8)	0.0216 (8)	0.0144 (8)
C2	0.0560 (9)	0.0682 (11)	0.0539 (10)	0.0155 (8)	0.0205 (8)	0.0195 (8)
C3	0.0518 (9)	0.0680 (11)	0.0534 (10)	0.0112 (8)	0.0188 (8)	0.0132 (8)
C4	0.0571 (9)	0.0697 (11)	0.0524 (10)	0.0097 (8)	0.0209 (8)	0.0179 (8)
C5	0.0624 (11)	0.0797 (12)	0.0653 (12)	0.0183 (9)	0.0176 (9)	0.0284 (10)
C6	0.0529 (9)	0.0805 (12)	0.0681 (12)	0.0178 (9)	0.0230 (9)	0.0253 (10)
C7	0.0606 (11)	0.0953 (14)	0.0749 (13)	0.0283 (10)	0.0218 (10)	0.0271 (11)
C8	0.0993 (16)	0.142 (2)	0.0828 (16)	0.0479 (15)	0.0419 (13)	0.0677 (16)
C9	0.0525 (9)	0.0718 (11)	0.0608 (11)	0.0147 (8)	0.0211 (8)	0.0171 (9)
C10	0.0560 (9)	0.0566 (10)	0.0512 (10)	0.0128 (7)	0.0181 (8)	0.0098 (7)
C11	0.0573 (9)	0.0574 (10)	0.0468 (9)	0.0136 (7)	0.0173 (7)	0.0134 (7)
C12	0.0574 (9)	0.0535 (9)	0.0502 (9)	0.0100 (7)	0.0213 (8)	0.0075 (7)
C13	0.0768 (12)	0.0540 (10)	0.0528 (10)	0.0082 (8)	0.0233 (9)	0.0149 (8)
C14	0.0854 (13)	0.0651 (11)	0.0590 (11)	0.0227 (9)	0.0169 (10)	0.0213 (9)
C15	0.0633 (10)	0.0695 (11)	0.0625 (11)	0.0218 (9)	0.0149 (9)	0.0175 (9)
O5	0.0773 (8)	0.0624 (7)	0.0604 (7)	0.0180 (6)	0.0350 (6)	0.0249 (6)
O6	0.1004 (9)	0.0575 (7)	0.0747 (8)	0.0342 (7)	0.0466 (7)	0.0314 (6)
O7	0.1593 (15)	0.0864 (10)	0.1150 (13)	0.0412 (10)	0.0760 (12)	0.0647 (10)
O8	0.1480 (14)	0.0737 (9)	0.0983 (12)	0.0427 (9)	0.0652 (11)	0.0337 (8)
N3	0.0721 (9)	0.0517 (8)	0.0526 (8)	0.0069 (7)	0.0223 (7)	0.0160 (6)
N4	0.0789 (10)	0.0673 (10)	0.0732 (11)	0.0208 (8)	0.0290 (9)	0.0341 (8)
C16	0.0634 (10)	0.0474 (9)	0.0478 (9)	0.0052 (7)	0.0166 (8)	0.0129 (7)
C17	0.1022 (14)	0.0473 (10)	0.0685 (12)	0.0185 (9)	0.0394 (11)	0.0166 (8)
C18	0.0878 (13)	0.0570 (10)	0.0609 (11)	0.0204 (9)	0.0349 (10)	0.0134 (8)
C19	0.0559 (9)	0.0537 (9)	0.0485 (9)	0.0097 (7)	0.0181 (8)	0.0153 (7)
C20	0.0560 (9)	0.0498 (9)	0.0507 (9)	0.0136 (7)	0.0157 (7)	0.0164 (7)
C21	0.0605 (9)	0.0531 (9)	0.0496 (9)	0.0142 (7)	0.0205 (8)	0.0141 (7)
C22	0.0904 (13)	0.0826 (13)	0.0715 (13)	0.0247 (11)	0.0485 (11)	0.0257 (10)
C23	0.1131 (16)	0.0639 (11)	0.0861 (14)	0.0428 (11)	0.0515 (13)	0.0273 (10)
C24	0.0638 (10)	0.0492 (9)	0.0559 (10)	0.0071 (7)	0.0170 (8)	0.0160 (8)
C25	0.0535 (9)	0.0561 (10)	0.0480 (9)	0.0059 (7)	0.0117 (7)	0.0143 (7)
C26	0.0574 (9)	0.0591 (10)	0.0483 (9)	0.0113 (8)	0.0173 (8)	0.0159 (8)
C27	0.0538 (9)	0.0614 (10)	0.0496 (9)	0.0114 (7)	0.0139 (7)	0.0204 (8)
C28	0.0650 (10)	0.0766 (13)	0.0535 (11)	0.0128 (9)	0.0224 (9)	0.0250 (9)
C29	0.0775 (12)	0.0793 (13)	0.0613 (12)	0.0184 (10)	0.0334 (10)	0.0155 (10)
C30	0.0711 (11)	0.0608 (11)	0.0612 (11)	0.0132 (9)	0.0225 (9)	0.0121 (9)

### Geometric parameters (Å, °)

**Table:**

O1—C3	1.363 (2)	C7—H7A	0.9600
O1—C7	1.418 (3)	C8—H8C	0.9600
O2—C4	1.365 (2)	C8—H8B	0.9600
O2—C8	1.404 (3)	C8—H8A	0.9600
O3—N2	1.218 (2)	C9—H9	0.9300
O4—N2	1.216 (2)	C11—H11	0.9300
O5—C22	1.427 (3)	C13—H13	0.9300
O5—C19	1.363 (2)	C14—H14	0.9300
O6—C23	1.422 (3)	C15—H15	0.9300
O6—C20	1.369 (2)	C16—C17	1.373 (3)
O7—N4	1.214 (2)	C16—C21	1.401 (2)
O8—N4	1.208 (2)	C17—C18	1.390 (3)
N1—C1	1.419 (2)	C18—C19	1.371 (2)
N1—C9	1.258 (2)	C19—C20	1.408 (2)
N2—C12	1.468 (2)	C20—C21	1.377 (2)
N3—C24	1.268 (2)	C24—C25	1.470 (2)
N3—C16	1.416 (2)	C25—C26	1.387 (2)
N4—C27	1.471 (2)	C25—C30	1.385 (2)
C1—C2	1.403 (2)	C26—C27	1.376 (2)
C1—C6	1.369 (2)	C27—C28	1.380 (2)
C2—C3	1.378 (2)	C28—C29	1.371 (2)
C3—C4	1.401 (2)	C29—C30	1.387 (3)
C4—C5	1.373 (3)	C17—H17	0.9300
C5—C6	1.389 (3)	C18—H18	0.9300
C9—C10	1.467 (2)	C21—H21	0.9300
C10—C15	1.391 (2)	C22—H22A	0.9600
C10—C11	1.390 (2)	C22—H22B	0.9600
C11—C12	1.376 (2)	C22—H22C	0.9600
C12—C13	1.380 (2)	C23—H23A	0.9600
C13—C14	1.366 (3)	C23—H23B	0.9600
C14—C15	1.383 (3)	C23—H23C	0.9600
C2—H2	0.9300	C24—H24	0.9300
C5—H5	0.9300	C26—H26	0.9300
C6—H6	0.9300	C28—H28	0.9300
C7—H7B	0.9600	C29—H29	0.9300
C7—H7C	0.9600	C30—H30	0.9300
			
O1···O2	2.575 (2)	C21···H23B	2.7600
O1···C22^i^	3.410 (3)	C22···H18	2.5300
O2···O1	2.575 (2)	C23···H26^v^	2.8400
O2···C22^i^	3.220 (3)	C23···H21	2.5000
O3···C7^ii^	3.373 (3)	C24···H21	2.6600
O4···C29^iii^	3.322 (2)	C28···H14^xii^	3.0600
O4···C28^iii^	3.222 (2)	C29···H23C^xiii^	3.0400
O4···C3^iv^	3.337 (2)	H2···H7B	2.2800
O5···C14^v^	3.206 (2)	H2···O3^x^	2.8500
O5···C30^vi^	3.285 (2)	H2···C7	2.4900
O5···O6	2.5677 (18)	H2···H7C	2.2900
O6···O5	2.5677 (18)	H2···H9	2.0600
O7···C8^vii^	3.268 (3)	H2···C9	2.6400
O8···C23^viii^	3.274 (3)	H5···C8	2.5100
O1···H22A^i^	2.6000	H5···H8B	2.2600
O2···H22C^i^	2.6400	H5···O5^ix^	2.8800
O3···H8B^ix^	2.7400	H5···H8C	2.3400
O3···H9^ii^	2.9100	H6···H7A^ii^	2.4900
O3···H11	2.4100	H7A···H6^x^	2.4900
O3···H7B^iv^	2.8000	H7B···H2	2.2800
O3···H2^ii^	2.8500	H7B···C2	2.7200
O3···H7C^ii^	2.8200	H7B···O3^iv^	2.8000
O4···H28^iii^	2.5700	H7C···H11^x^	2.5700
O4···H13	2.4400	H7C···O3^x^	2.8200
O4···H29^iii^	2.7600	H7C···C2	2.7300
O5···H14^v^	2.5500	H7C···H2	2.2900
O5···H5^ix^	2.8800	H7C···C5^i^	2.9900
O6···H13^v^	2.8000	H7C···C6^i^	2.8700
O7···H28	2.4400	H8A···O7^xi^	2.3900
O7···H30^viii^	2.8900	H8A···H22C^i^	2.5100
O7···H8A^vii^	2.3900	H8B···C5	2.7200
O8···H23C^viii^	2.7100	H8B···H5	2.2600
O8···H24^viii^	2.8800	H8B···O3^ix^	2.7400
O8···H21^viii^	2.7500	H8C···C19^ix^	3.0200
O8···H26	2.4400	H8C···C5	2.7500
N2···C2^iv^	3.403 (2)	H8C···H5	2.3400
N2···C3^iv^	3.389 (2)	H8C···C20^ix^	2.7700
N4···C24^iii^	3.380 (2)	H9···H15	2.4800
N1···H11	2.5700	H9···O3^x^	2.9100
N3···H23B^vi^	2.8000	H9···C2	2.5400
N3···H23A^viii^	2.9500	H9···H2	2.0600
N3···H26	2.5700	H11···C7^ii^	2.9900
C2···N2^iv^	3.403 (2)	H11···H7C^ii^	2.5700
C2···C3^i^	3.594 (2)	H11···O3	2.4100
C3···C2^i^	3.594 (2)	H11···N1	2.5700
C3···O4^iv^	3.337 (2)	H13···O6^viii^	2.8000
C3···N2^iv^	3.389 (2)	H13···O4	2.4400
C7···O3^x^	3.373 (3)	H14···O5^viii^	2.5500
C8···O7^xi^	3.268 (3)	H14···C28^xii^	3.0600
C13···C18	3.598 (2)	H15···H9	2.4800
C14···O5^viii^	3.206 (2)	H17···C13	3.0900
C18···C13	3.598 (2)	H17···H23A^viii^	2.2800
C19···C30^vi^	3.504 (2)	H18···C13	3.0800
C20···C24^vi^	3.341 (2)	H18···C22	2.5300
C20···C25^vi^	3.552 (2)	H18···H22B	2.3000
C21···C24^vi^	3.498 (2)	H18···H22C	2.3500
C22···O2^i^	3.220 (3)	H21···O8^v^	2.7500
C22···O1^i^	3.410 (3)	H21···C23	2.5000
C23···O8^v^	3.274 (3)	H21···C24	2.6600
C24···C21^vi^	3.498 (2)	H21···H23B	2.3600
C24···C27^iii^	3.599 (2)	H21···H23C	2.2200
C24···N4^iii^	3.380 (2)	H21···H24	2.0500
C24···C20^vi^	3.341 (2)	H22A···O1^i^	2.6000
C25···C20^vi^	3.552 (2)	H22B···C11	3.0100
C25···C26^iii^	3.582 (2)	H22B···C18	2.7500
C26···C25^iii^	3.582 (2)	H22B···H18	2.3000
C26···C26^iii^	3.403 (2)	H22C···C18	2.7700
C27···C24^iii^	3.599 (2)	H22C···H18	2.3500
C28···O4^iii^	3.222 (2)	H22C···O2^i^	2.6400
C29···O4^iii^	3.322 (2)	H22C···C8^i^	3.0300
C30···C19^vi^	3.504 (2)	H22C···H8A^i^	2.5100
C30···O5^vi^	3.285 (2)	H23A···N3^v^	2.9500
C2···H7C	2.7300	H23A···C17^v^	3.1000
C2···H7B	2.7200	H23A···H17^v^	2.2800
C2···H9	2.5400	H23B···C21	2.7600
C5···H7C^i^	2.9900	H23B···H21	2.3600
C5···H8B	2.7200	H23B···N3^vi^	2.8000
C5···H8C	2.7500	H23C···O8^v^	2.7100
C6···H7C^i^	2.8700	H23C···C21	2.6900
C7···H2	2.4900	H23C···H21	2.2200
C7···H11^x^	2.9900	H23C···C29^xiii^	3.0400
C8···H5	2.5100	H24···O8^v^	2.8800
C8···H22C^i^	3.0300	H24···C21	2.5500
C9···H2	2.6400	H24···H21	2.0500
C11···H22B	3.0100	H24···H30	2.4600
C13···H17	3.0900	H26···O8	2.4400
C13···H18	3.0800	H26···N3	2.5700
C17···H23A^viii^	3.1000	H26···C23^viii^	2.8400
C18···H22C	2.7700	H28···O7	2.4400
C18···H22B	2.7500	H28···O4^iii^	2.5700
C19···H8C^ix^	3.0200	H29···O4^iii^	2.7600
C20···H8C^ix^	2.7700	H30···O7^v^	2.8900
C21···H24	2.5500	H30···H24	2.4600
C21···H23C	2.6900		
			
C3—O1—C7	117.12 (14)	C14—C13—H13	121.00
C4—O2—C8	117.65 (16)	C12—C13—H13	121.00
C19—O5—C22	117.37 (13)	C13—C14—H14	120.00
C20—O6—C23	116.66 (14)	C15—C14—H14	120.00
C1—N1—C9	121.85 (15)	C14—C15—H15	119.00
O3—N2—O4	123.16 (17)	C10—C15—H15	119.00
O4—N2—C12	118.00 (16)	N3—C16—C17	116.25 (14)
O3—N2—C12	118.84 (16)	N3—C16—C21	125.11 (15)
C16—N3—C24	121.57 (14)	C17—C16—C21	118.65 (15)
O7—N4—O8	122.33 (17)	C16—C17—C18	121.30 (16)
O7—N4—C27	118.52 (16)	C17—C18—C19	120.26 (16)
O8—N4—C27	119.14 (16)	O5—C19—C18	125.71 (15)
N1—C1—C2	124.00 (15)	O5—C19—C20	115.11 (14)
N1—C1—C6	117.13 (15)	C18—C19—C20	119.18 (15)
C2—C1—C6	118.83 (16)	O6—C20—C19	115.11 (14)
C1—C2—C3	120.08 (16)	O6—C20—C21	124.80 (15)
O1—C3—C2	124.52 (16)	C19—C20—C21	120.09 (15)
C2—C3—C4	120.24 (16)	C16—C21—C20	120.52 (16)
O1—C3—C4	115.23 (15)	N3—C24—C25	121.11 (15)
O2—C4—C3	115.77 (15)	C24—C25—C26	121.08 (15)
C3—C4—C5	119.56 (16)	C24—C25—C30	120.06 (15)
O2—C4—C5	124.67 (16)	C26—C25—C30	118.85 (16)
C4—C5—C6	119.80 (17)	C25—C26—C27	119.36 (16)
C1—C6—C5	121.48 (16)	N4—C27—C26	118.80 (15)
N1—C9—C10	121.83 (16)	N4—C27—C28	118.86 (15)
C11—C10—C15	118.52 (16)	C26—C27—C28	122.34 (16)
C9—C10—C11	120.57 (15)	C27—C28—C29	118.05 (16)
C9—C10—C15	120.89 (16)	C28—C29—C30	120.76 (17)
C10—C11—C12	118.74 (16)	C25—C30—C29	120.65 (16)
C11—C12—C13	122.91 (16)	C16—C17—H17	119.00
N2—C12—C11	117.96 (15)	C18—C17—H17	119.00
N2—C12—C13	119.11 (15)	C17—C18—H18	120.00
C12—C13—C14	118.19 (16)	C19—C18—H18	120.00
C13—C14—C15	120.30 (17)	C16—C21—H21	120.00
C10—C15—C14	121.31 (16)	C20—C21—H21	120.00
C3—C2—H2	120.00	O5—C22—H22A	109.00
C1—C2—H2	120.00	O5—C22—H22B	109.00
C4—C5—H5	120.00	O5—C22—H22C	109.00
C6—C5—H5	120.00	H22A—C22—H22B	110.00
C5—C6—H6	119.00	H22A—C22—H22C	109.00
C1—C6—H6	119.00	H22B—C22—H22C	109.00
O1—C7—H7C	109.00	O6—C23—H23A	109.00
O1—C7—H7A	109.00	O6—C23—H23B	109.00
O1—C7—H7B	109.00	O6—C23—H23C	110.00
H7B—C7—H7C	109.00	H23A—C23—H23B	109.00
H7A—C7—H7B	109.00	H23A—C23—H23C	109.00
H7A—C7—H7C	110.00	H23B—C23—H23C	109.00
O2—C8—H8A	109.00	N3—C24—H24	119.00
O2—C8—H8C	109.00	C25—C24—H24	119.00
H8A—C8—H8B	109.00	C25—C26—H26	120.00
O2—C8—H8B	110.00	C27—C26—H26	120.00
H8B—C8—H8C	109.00	C27—C28—H28	121.00
H8A—C8—H8C	109.00	C29—C28—H28	121.00
N1—C9—H9	119.00	C28—C29—H29	120.00
C10—C9—H9	119.00	C30—C29—H29	120.00
C10—C11—H11	121.00	C25—C30—H30	120.00
C12—C11—H11	121.00	C29—C30—H30	120.00
			
C7—O1—C3—C2	−1.0 (3)	N1—C9—C10—C15	174.86 (17)
C7—O1—C3—C4	179.84 (17)	C11—C10—C15—C14	1.0 (3)
C8—O2—C4—C5	−3.2 (3)	C9—C10—C15—C14	−177.38 (17)
C8—O2—C4—C3	176.40 (17)	C15—C10—C11—C12	0.2 (2)
C22—O5—C19—C18	−2.8 (2)	C9—C10—C11—C12	178.56 (16)
C22—O5—C19—C20	177.79 (16)	C10—C11—C12—C13	−1.5 (3)
C23—O6—C20—C21	6.2 (3)	C10—C11—C12—N2	177.18 (15)
C23—O6—C20—C19	−174.41 (17)	N2—C12—C13—C14	−177.09 (16)
C1—N1—C9—C10	−176.43 (16)	C11—C12—C13—C14	1.5 (3)
C9—N1—C1—C6	−170.48 (17)	C12—C13—C14—C15	−0.3 (3)
C9—N1—C1—C2	11.8 (3)	C13—C14—C15—C10	−0.9 (3)
O4—N2—C12—C13	3.4 (2)	C17—C16—C21—C20	0.0 (3)
O3—N2—C12—C13	−176.91 (17)	C21—C16—C17—C18	−0.4 (3)
O3—N2—C12—C11	4.4 (2)	N3—C16—C21—C20	−179.64 (16)
O4—N2—C12—C11	−175.31 (17)	N3—C16—C17—C18	179.29 (16)
C24—N3—C16—C21	−6.9 (3)	C16—C17—C18—C19	0.2 (3)
C16—N3—C24—C25	179.68 (16)	C17—C18—C19—C20	0.4 (3)
C24—N3—C16—C17	173.53 (17)	C17—C18—C19—O5	−178.99 (16)
O8—N4—C27—C28	173.22 (18)	C18—C19—C20—O6	179.79 (15)
O7—N4—C27—C28	−6.1 (3)	O5—C19—C20—C21	178.67 (15)
O7—N4—C27—C26	174.45 (18)	O5—C19—C20—O6	−0.7 (2)
O8—N4—C27—C26	−6.2 (3)	C18—C19—C20—C21	−0.8 (3)
N1—C1—C6—C5	−178.02 (16)	C19—C20—C21—C16	0.6 (3)
C6—C1—C2—C3	−0.1 (3)	O6—C20—C21—C16	179.96 (15)
N1—C1—C2—C3	177.54 (16)	N3—C24—C25—C26	0.6 (3)
C2—C1—C6—C5	−0.2 (3)	N3—C24—C25—C30	−178.79 (17)
C1—C2—C3—O1	−178.89 (16)	C24—C25—C26—C27	−179.43 (16)
C1—C2—C3—C4	0.2 (3)	C30—C25—C26—C27	−0.1 (2)
C2—C3—C4—O2	−179.55 (16)	C26—C25—C30—C29	0.2 (3)
O1—C3—C4—O2	−0.4 (2)	C24—C25—C30—C29	179.50 (16)
C2—C3—C4—C5	0.1 (3)	C25—C26—C27—N4	179.48 (16)
O1—C3—C4—C5	179.23 (16)	C25—C26—C27—C28	0.1 (3)
O2—C4—C5—C6	179.20 (17)	N4—C27—C28—C29	−179.54 (16)
C3—C4—C5—C6	−0.4 (3)	C26—C27—C28—C29	−0.1 (3)
C4—C5—C6—C1	0.5 (3)	C27—C28—C29—C30	0.2 (3)
N1—C9—C10—C11	−3.5 (3)	C28—C29—C30—C25	−0.2 (3)

Symmetry codes: (i) −*x*+1, −*y*+1, −*z*; (ii) *x*−1, *y*, *z*; (iii) −*x*−1, −*y*, −*z*+1; (iv) −*x*, −*y*, −*z*; (v) *x*, *y*+1, *z*; (vi) −*x*, −*y*+1, −*z*+1; (vii) *x*−1, *y*−1, *z*+1; (viii) *x*, *y*−1, *z*; (ix) −*x*, −*y*+1, −*z*; (x) *x*+1, *y*, *z*; (xi) *x*+1, *y*+1, *z*−1; (xii) −*x*, −*y*, −*z*+1; (xiii) −*x*−1, −*y*+1, −*z*+1.

### Hydrogen-bond geometry (Å, °)

**Table:**

*D*—H···*A*	*D*—H	H···*A*	*D*···*A*	*D*—H···*A*
C8—H8A···O7^xi^	0.96	2.39	3.268 (3)	152
C14—H14···O5^viii^	0.93	2.55	3.206 (2)	128
C22—H22A···O1^i^	0.96	2.60	3.410 (3)	143
C28—H28···O4^iii^	0.93	2.57	3.222 (2)	128
C18—H18···Cg1	0.93	2.89	3.654 (2)	141

Symmetry codes: (xi) *x*+1, *y*+1, *z*−1; (viii) *x*, *y*−1, *z*; (i) −*x*+1, −*y*+1, −*z*; (iii) −*x*−1, −*y*, −*z*+1.
